# Oncology Healthcare Professionals’ Mental Health during the COVID-19 Pandemic

**DOI:** 10.3390/curroncol29060323

**Published:** 2022-06-02

**Authors:** Leeat Granek, Ora Nakash

**Affiliations:** 1 School of Health Policy and Management and Department of Psychology, Faculty of Health, York University, 4700 Keele Street, Toronto, ON M3J 1P3, Canada; 2 School for Social Work, Smith College, Northampton, MA 01063, USA; onakash@smith.edu

**Keywords:** oncology, oncologists, oncology nurses, COVID-19 pandemic, depression, burnout, anxiety

## Abstract

The paper begins by reviewing the literature on oncology healthcare professionals’ (HCP) mental health. We summarize and present the current data on HCP mental health in order to understand the baseline state of oncology HCPs’ mental health status *prior* to the COVID-19 pandemic. At each juncture, we will discuss the implications of these mental health variables on the personal lives of HCPs, the healthcare system, and patient care. We follow by reviewing the literature on these parameters during the COVID-19 pandemic in order to better understand the impact of COVID-19 on the overall mental health of HCPs working in oncology. By reviewing and summarizing the data before and after the start of the pandemic, we will get a fuller picture of the pre-existing stressors facing oncology HCPs and the added burden caused by pandemic-related stresses. The second part of this review paper will discuss the implications for the oncology workforce and offer recommendations based on the research literature in order to improve the lives of HCPs, and in the process, improve patient care.

## 1. Introduction

We started writing this article as we entered year three of the COVID-19 pandemic during the heart of the Omicron surge in December 2021. In North America, where we both reside, and across the globe, hospitals were once again at full capacity and ICU beds and healthcare professionals caring for COVID-19 patients were stretched thin. As a result of eroding resources which were now almost entirely dedicated to caring for patients with COVID-19, all non-elective surgeries were cancelled, and in-person clinic visits in other professions, including oncology, were minimized to prevent spread. This fifth wave of the pandemic and its reverberating effects on all medical specialties coincided with my (Granek) project putting together this Special Issue for *Current Oncology* focused on the well-being of healthcare professionals (HCPs) in oncology. While a lot has been written about the mental health of front-line workers caring for patients with COVID-19 in hospitals and other acute care settings [1,2,3,4,5], less has been written about the oncology workforce during this time. As such, the focus of this review paper is on the impact of the COVID-19 pandemic on oncology HCPs’ mental health. 

In order to understand the impact of the pandemic on oncologists, nurses, and other oncology HCPs, it is necessary to first understand their baseline mental health risks and status prior to the start of the pandemic. As such, we begin this paper by reviewing the literature on oncology HCPs’ mental health *prior* to the start of the pandemic. At each juncture, we discuss the implications of these mental health variables on the personal lives of HCPs, the healthcare system, and patient care. We then review the literature on these parameters during the COVID-19 pandemic in order to better understand the impact of COVID-19 on the overall mental health of HCPs working in oncology. We conclude this paper by focusing on the implications of the COVID-19 pandemic for the oncology workforce and offer recommendations based on the research literature in order to improve the lives of HCPs, and in the process, improve patient care.

## 2. Methodology

***Sic:***

### 2.1. Search 1—Oncology Healthcare Professionals’ (HCP) Mental Health Pre-Pandemic

The goal of this search was to provide a summary of the research before the COVID-19 pandemic on oncology HCPs’ burnout, grief responses, mental health (i.e., anxiety and depression, post-traumatic stress disorder (PTSD), alcohol use, substance use, suicidality), and moral distress. We used PubMed, Medline, and Google Scholar databases to identify relevant articles using the following key words: ‘oncolo*’ AND general terms to identify mental health and related issues that included the following search terms: ‘mental health’; ‘psychiatric disorder’; ‘emotional distress’; ‘burnout’; ‘moral distress’; ‘moral injury’; ‘grief’ as well as specific psychopathology that included the following search terms: depression’; ‘anxiety’; ‘PTSD’; ‘suicid*’; ‘substance use’; ‘alcohol’. Finally, to narrow the search we added the keywords ‘healthcare provider’ or ‘physician’ or ‘nurse’ or ‘psychosocial clinician’ or ‘social work’ or ‘psycholog*’. The search was limited to papers that reported on data collected prior to 2019 and all the searches were run in January 2022. While an overwhelming number of papers have been published on *patient* mental health concerns, far fewer studies have examined HCPs’ mental health status. Of the studies published on oncology HCPs, the overwhelming majority focused on burnout, moral distress, and grief reactions. Only a handful of studies examined mental health, suicidality, and substance use. In total, we included 54 papers in this review and prioritized recent reviews and meta-analyses for topics such as burnout that have received more attention in the literature.

### 2.2. Search 2—Impact of COVID-19 Pandemic on Oncology Healthcare Professionals’ Mental Health

The goal of the second search was to identify articles published on the impact of the COVID-19 pandemic on oncology HCPs’ mental health. The following electronic databases were used: Medline, PsychInfo, Embase, CINAHL, PubMed, and Google Scholar. We used the following key words: ‘oncolo*’ OR ‘oncol* nurse’ OR oncolo* healthcare provider’ AND COVID-19 or SARS-CoV-2 OR pandemic AND ‘mental health’; ‘burnout’; ‘moral distress’; ‘moral injury’; ‘distress’; depression’; ‘anxiety’; ‘suicid*; ‘grief’; ‘substance use’; ‘alcohol’ as well as a more targeted search adding the keywords ‘healthcare provider’; ‘physician’; ‘nurse’; ‘psychosocial clinician.’ The search was limited from 2020 to 2022 and all the searches were run in March 2022. The second approach to this inquiry involved ‘snowball searching’ on PubMed and Google Scholar. Relevant article titles were entered into the search box on PubMed and Google Scholar and the ‘show similar papers’ search button was utilized. Eligible studies included studies looking at the impact of COVID-19 on any type of oncology healthcare provider. In total, 24 eligible articles were included and were reviewed for themes discussed in more detail below.

## 3. Mental Health of Oncology HCPs *Prior to* the COVID-19 Pandemic

*t:*

The results of our search are summarized in Table 1 and presented in more detail below. 

### 3.1. Burnout

Oncology HCPs are at a particularly high risk of burnout. Burnout is a syndrome that results from continuous exposure to occupational stressors and consists of three dimensions: high emotional exhaustion, high depersonalization, and low personal accomplishment [6]. Oncology healthcare providers’ daily experiences include caring for patients who are suffering from cancer and its outcomes and dealing with patients who may be nearing the end of life. Thus, these HCPs often need to cope with their own grief over losing patients, as well as their patient’s and caregivers’ grief over losses associated with the illness, deliver difficult news, and make ethically complex decisions. Physicians have rated oncology as one of the most stressful specialties generating high levels of emotional exhaustion [7]. A recent systematic review that pooled prevalence rates of burnout among 5768 oncologists showed that approximately a third of oncologists experience burnout [8]. Specifically, 32% reported emotional exhaustion, 24% reported depersonalization, and 37% reported low personal accomplishment [8]. Other burnout studies have documented a range of prevalence rates. Specifically, approximately a third of medical oncologists (25–35%) and surgical oncologists (28–36%), and a quarter of radiation oncologists (28%) report burnout [9]. Female oncologists seem to be at an elevated risk of burnout [10,11]. Some authors have drawn attention to the risk of compassion fatigue, that is defined by burnout and secondary traumatic stress and can result in reduced interest in patients and reduced empathy towards their distress. In a recent study among oncologists, compassion fatigue was associated with subjective experiences of grief and feelings of professional failure [12].

Similar high rates of burnout have been documented among oncology nurses [13]. In a recent meta-analysis that pooled data on close to 10,000 oncology nurses, 30% reported emotional exhaustion, 15% reported depersonalization, and 35% reported low personal performance [14]. Similar rates have been documented in a meta-analysis on burnout among pediatric oncology nurses, where 31% reported emotional exhaustion, 21% reported depersonalization, and 39% reported low personal accomplishment [15]. Psychosocial clinicians also seem to be at risk of burnout. For example, a recent systematic review on burnout among psychosocial clinicians working in oncology settings (e.g., social workers, psychologists) showed that they had a similar high risk of burnout as other oncology HCPs as well as mental health clinicians working in other health disciplines [16].

Risk factors for burnout include chronic exposure to patient death and challenges of end-of-life care [17]; heavy workload and time pressures [18]; under-resourced work environments and ineffective management [19]; difficulty managing demands of work and family [8]; and loss of meaning at work [19]. Moreover, qualitative studies documented that oncologists’ grief following a patient death can result in increased emotional distress and burnout [20,21]. Experiences of burnout among oncology HCPs can impact their work satisfaction [9,22], as well as lead to turnover [23]. It can negatively affect the quality of patient care [24], increase medical errors [25], and result in significant financial losses for the healthcare system [26].

### 3.2. Grief

The chronic exposure to patient death necessitates that HCPs not only support the patient and their caregivers in their grieving processes, but that they also attend to their own responses to patient loss. Studies in the last decade have examined the experiences of oncology HCPs following a loss of a patient [20,27,28,29,30]. A study of oncologists found that patient death could lead to experiences of emotional exhaustion, challenges in holding emotional boundaries between work and life, and feeling as if this grief affected the oncologists’ personal relationships negatively. The impact of patient loss on HCPs’ professional behaviors and decisions included focusing on active treatment instead of turning to palliative care, and the tendency to withdraw from patients when they were nearing death [20]. An additional study that examined pediatric oncologists’ experiences following a patient death documented increased irritability at home, feeling disconnected from personal connections (e.g., family friends), and becoming more desensitized to death [29]. Female oncologists tend to report significantly more emotional distress and grief responses to patient loss compared with their male counterparts [10]. Despite the importance of supporting oncology HCPs in their grieving processes, many report receiving minimal institutional help and formal training to deal with patient loss [31,32].

Studies among oncology nurses have also documented grief responses to patient loss and have identified factors that may exacerbate experiences of grief. These factors include high workloads which leaves little time for respite or recreational breaks, limited support in and outside the hospital, as well and difficulties communicating with personnel outside the nursing staff [33,34]. In a review of the literature, oncology nurses reported similar concerns around a lack of education and training related to bereavement care and experiences of inadequacy in delivering quality end-of-life care, which was often related to staffing shortages. Importantly, oncology nurses often suppress explicit expression of grief in the presence of patients, families, and peers, considering such behaviors unprofessional and often leaving them to deal with these difficult experiences in isolation [33].

### 3.3. Psychiatric Morbidity (Emotional Distress, Depression, Anxiety, Substance Use, and Suicidality)

Although limited in scope, some studies have focused on psychiatric morbidity among oncology HCPs. Most notably these studies focused on common mental disorders such as stress, anxiety, and depression [35]. Explicit examination of oncology HCPs’ mental health syndromes have historically received less attention, partly as a result of stigma and overvaluing of stoic approach to manage hardship in medical education [36]. A recent review documented that oncologists tend to report higher rates of depression than their internal medicine colleagues [36]. In a study of 1436 Japanese clinical oncologists and palliative care physicians, approximately 20% of respondents reported psychiatric morbidity (i.e., depression and anxiety). Clinical oncologists reported significantly higher rates of depression and anxiety when compared to palliative care physicians [37]. In a different study among oncologists in the United States, 12% met criteria for depression and 19% for anxiety [38]. High workload and relationship problems with work peers were significantly associated with higher anxiety rates [38]. An additional study among surgical oncologists found that 27% of the sample reported high levels of mental health distress (i.e., depression and anxiety), 30% excessively used alcohol, and 13% reported using sleep medications to cope with work-related distress [39]. A recent meta-analysis that combined data on 4876 oncologists documented that 27% have high psychiatric morbidity, 42–69% tend to feel stressed at work, and approximately 12% screen positive for depression, with sleep deprivation being a common symptom. Furthermore, 30% drink alcohol excessively, 20% of junior oncologists use hypnotic drugs (i.e., medications used to improve the quality of sleep such as anti-anxiety, antidepressants, and antihistamines) and some reported stress-induced complaints such as gastric problems and headaches [40].

Although limited research exists, some studies have also documented high suicide rates among physicians, including oncologists [41]. Oncologists are particularly at risk for emotional distress and potentially suicidality because of the taxing nature of their work and the emotional toll of depressive symptoms in reaction to potential exposure to vicarious trauma in the context of caring for terminally ill patients [36].

### 3.4. Moral Distress and Moral Injury

Oncology HCPs may struggle with many ethical challenges in end-of-life care. In recent decades, there has been growing attention to the moral distress of oncology HCPs as it is considered a root cause for providers’ burnout and psychiatric morbidity [42]. Although definitions for moral distress vary, in 1984, Jameton suggested that “Moral distress arises when one knows the right thing to do, but institutional constraints make it nearly impossible to pursue the right course of action” [43] p. 6. Some of these institutional constraints include policies and situations that make it challenging for clinicians to maintain the values they honor, as well as internal and external barriers to behaving according to their ethical values (e.g., concerns around professional admonishment, legal limitations and coercion, healthcare setting policies and procedures) [44].

In a study among Swedish pediatric oncology healthcare providers, participants stated that contributors to moral distress included not having time to adequately communicate with patients and caregivers and limited ability to meet the expectations of caretaking given the constraints of the system [45]. Another study among Swedish pediatric oncology HCPs documented that incompetence among HCPs and personnel turnover were ranked with the highest moral distress scores [46]. Additionally, while HCPs from all disciplines reported high levels of distress, nurses compared to physicians had significantly higher reports of moral distress [46]. A review of the literature on factors fostering experience of moral distress among nurses in oncology settings highlighted organizational factors (e.g., ethical climate, incoherent, and/or limited resources), specific clinical incidents (e.g., palliative care, failed treatment, and end-of-life care), and events related to interpersonal relationships (e.g., communication problems, power relations, lack of trust in competency of the HCP). Few studies examined the impact of prolonged experiences of moral distress. Studies, particularly among nurses, documented that moral distress can result in increased emotional distress and burnout and reduced job satisfaction and retention in the profession [47,48]. A recent review of the literature found that persistent experiences of moral distress can lead to diminished moral sensitivity, increased psychological and health problems, and increased turnover intention [49].

## 4. Impact of COVID-19 Pandemic on Oncology Healthcare Professionals’ Mental Health

“I think that all of our colleagues are feeling a lot of burnout right now. Everybody’s seeing a lot of death and heartache and social isolation and anger that they’re not used to encountering in very new and different ways” [50] p. 431.

The results of our search are summarized in Table 2. In reviewing the literature on oncology HCPs’ mental health during the pandemic, a few important factors became immediately apparent. The first is that most of the studies published were conducted during the *first wave* of COVID-19. Studies that took a longer view of the impact of the pandemic on oncology HCPs found that mental health distress increased substantially and alarmingly over relatively short periods of time [51]. For example, one study that looked at the concerns of oncology healthcare professionals during the COVID-19 pandemic found that over a three month period there was a substantial increase in burnout (49% versus 38%) [51]. This suggests that there is a compounding and accumulating effect of pandemic stress and that even a few months of coping with this situation substantially deceased oncology HCPs’ mental health. This point is one we will return to in the discussion; however, it is important to note here that the long-term nature of the pandemic has likely increased the burden on oncology HCPs and will have long-term impacts that will need to be addressed even if COVID-19 eventually recedes. The second important point to consider is that the COVID-19 pandemic has evolved rapidly and information about the airborne nature of the illness, vaccines for HCPs and their families, and new treatments have emerged over the past few years and thus many HCPs are struggling to keep up with the ever-changing public health guidelines. Stressors that HCPs worried about during the first wave when most of the research was conducted will have likely changed with new developments. For example, HCPs who were worried about being infected during the first wave when there were no vaccines available might be more concerned now about delayed treatments for their patients. The research we reviewed articulated *all* the concerns and impacts of the COVID-19 pandemic on oncology HCPs, but this research does not capture the trajectory and fluidity of these worries and impacts as the virus evolves and as public health guidelines change.

## 5. Rapid Changes to the Nature of HCPs’ Work and Day-to-Day Lives

### 5.1. Changes at Work

All of the studies reviewed noted that there were rapid and sudden changes to the nature of oncology HCPs’ day-to-day work lives including a dramatic decrease in face-to-face interactions with patients [52,53], deployment to other area of the hospitals [54] (including working on the front-line), lack of access to resources including personal protective equipment (PPE) [50,55], cancellation of surgeries and other treatments critical to patient’s health resulting in delays of live-saving treatments for patients [50], a substantially increased workload [54], loss of autonomy [54], reduced job security [51], reduced income [56], and reduction in research activities [56,57,58]. Moreover, oncologists were forced to transition to modifications in workflow schedules and to rapidly turn to virtual and telemedicine technologies [50].

### 5.2. Changes at Home

The pandemic also forced substantial changes to oncology HCPs’ home life; for some, this meant children at home doing online school, and for others this meant not seeing their children or their elderly parents for long stretches of time for fear of passing on the infection to them [53]. For example, one study that looked at social distress among medical oncologists and other oncology HCPs during the first wave of the pandemic in Italy found that a third of the respondents had not seen their children for more than two weeks and that more than 80% had not seen their elderly parents in this time frame [55]. Other HCPs reported that the quality of their relationships with their family had decreased as a result of the pandemic [56] and that they had inadequate time to spend with their families and/or attend to personal care at home [59]. One longitudinal survey of 1269 oncologists from 104 countries found that almost half of the respondents (45%) reported not having enough time for their families as a result of the pandemic and that this phenomenon had increased substantially from the first time data were collected from this sample early in the pandemic (34.6%) [59]. Another study that looked at the impact of the pandemic on male and female oncologists found that in a sample of 541 oncologists, females reported that the COVID-19 pandemic affected their personal life (89% vs. 78% of men) and family life (84% vs. 77% of men) in negative ways [60]. Similarly, a study of Indian physicians that included surgical and medical oncologists found that female HCPs had increased domestic responsibilities such as household chores and were more likely to be responsible for managing their child’s education when compared to their male colleagues. These changes at home caused them to feel that the pandemic was having an adverse impact on their professional work [61]. On a positive note, these changes also led some HCPs to develop a changed outlook on work–life balance as a result of the pandemic [50,51,62].

## 6. Impact of Pandemic-Related Changes on HCPs’ Mental Health

### 6.1. Psychological and Emotional Distress

The majority of studies published noted a marked increase in psychological distress among oncology HCPs [50,54,56,58,63,64], particularly high levels of anxiety [50,52,58,62,64,65,66]. Similar to the statistics prior to the pandemic, being young (under the age of 40) and female were risk factors for higher levels of emotional distress [59]. Oncology HCPs reported feeling anxiety about: their own health and contracting COVID-19 [50,52,55,56,66], about infecting their families with COVID-19 [50,53,55,56,65], about their lack of training and/or clear protocols about how to manage patients with COVID-19 [55], about their patient’s well-being, particularly those whose care was delayed as a result of the pandemic [53,66,67], and anxiety that the pandemic will go on for a long time [53]. One study that looked at 870 oncologists found that nearly 80% reported a delay in care for their patients as a result of the pandemic, and that this delay was correlated with high anxiety and burnout scores among physicians when compared to physicians whose patients did not experience treatment delays [67]. Another study that looked at emotional concerns of oncology physicians in the United States found that anxiety and depression were related to the inability to provide adequate care to patients with cancer [66].

Oncology HCPs also experienced feelings of a lack of control and uncertainty [51,52,64], guilt about not looking after their families, or providing the best care for their patients, feelings of depression [50,53,58,64,65,66], irritability [50], anger [50], post-traumatic stress symptoms ([63,64,68], peritraumatic distress [63], and sleep disturbance [67]. As a result of this distress, they reported using substances more frequently and noted an increase in the use of antidepressants and anti-anxiety medications [58]. For example, one study of oncology residents in France found that consumption of tobacco increased by 31%, psychostimulants by 24%, and alcohol 29% as a result of the pandemic [58].

### 6.2. Burnout, Moral Distress, and Moral Injury

In addition to the tremendous psychological burden on oncology HCPs as a result of the pandemic, they also reported high levels of burnout, moral distress, and moral injury. Increased burnout was robustly reported by oncologists around the globe during the pandemic [51,53,64,69,70,71]. For example, one study in Egypt found that nearly 80% of the oncologists contacted reported being more overwhelmed and burned out than they were before the pandemic [71]. Another study that looked at 942 oncology professionals from 99 countries found that nearly 50% reported burnout [51]. Similarly, research on pediatric oncology providers in the United States found that 52% of their sample (of 252 participants) reported high levels of burnout, and 48% reported low levels as a result of the pandemic [53].

Moral distress and moral injury were also substantial issues faced by oncology HCPs during the pandemic. As noted in the section on psychological distress, HCP depression and anxiety was directly correlated to being unable to provide what they perceived to be adequate patient care as a result of pandemic restrictions [66,67]. In a commentary on oncologists well-being, Hlubocky and colleagues hypothesized that the major sources of moral distress for oncologists during the pandemic included changes in patient treatment plans, balancing the risk of getting COVID-19 while providing cancer care, lack of competency in being able to manage COVID-19 in their patients with cancer, and potential disagreement with institutional policies around how to best manage patient care during the pandemic [62]. Finally, another qualitative study of 18 oncologists reported that regret and fear of failing patients were common feelings around the care provided during the pandemic. Oncologists worried that they did not do enough for their patients or did not do the ‘right thing’ for them [52].

## 7. Discussion

Reviewing the literature on oncology HCPs’ mental health before and after the pandemic revealed some interesting and important patterns in the way that HCPs’ mental health is understood within the field of medicine. In the discussion below, we highlight the implications from this review and suggest interventions that may assist in improving the mental health of oncology HCPs now and in the future.

### 7.1. From Individual Responsibility to a Collective Burden: The Case of Burnout among Oncology HCPs

Our review found that many studies on HCPs both pre- and post-pandemic have focused on burnout. One potential reason that burnout research is so popular on this population may be because it centers on occupational health, and thus may be less stigmatizing than focusing on the mental health of HCPs. The focus on occupational health rather than on individual HCP’s mental health moves the problem and the solutions to the structural conditions of practicing oncology, rather than on the individual physician. We see this as a positive development in the HCP literature since many studies have found that a quarter to three quarters of oncology HCPs report feeling burned out and that this issue has become worse as a result of the pandemic [72,73,74]. These high numbers suggest that the factors that contribute to burnout are widespread and have to do with the nature of the work and environmental concerns, rather than the problem of individual HCPs and that changes are needed *within* the profession to help reduce this problem. A recently published editorial (accepted just before the COVID-19 pandemic) looking at physician wellness, burnout, and moral distress among oncology HCPs noted that factors that contribute to burnout include increased time in direct patient care, exposure to suffering and death, high occupational demands, lack of control and flexibility, increased administrative duties, use of electronic medical records, increased workload due to high turnover, work–life balance, lack of support, and loss of altruism [50]. The authors proposed several interventions to help reduce burnout that include environmental interventions to reduce stress such as streamlining workflow and reducing administrative burdens, changing the culture and ethics of work, fostering engagements with colleagues, broadening clinical education resources on burnout, establishing clinician well-being and burnout as quality metrics for oncology practices, and providing resources [50]. We agree with these recommendations and see them as an important first step in improving oncology HCPs’ quality of life. Burnout is a critical factor to consider when it comes to HCP well-being both pre- and post-pandemic, and as noted above, much has been written on how to reduce burnout among oncology HCPs [8,25,36,50,74]. However, our review also found gaps in our understanding and knowledge on the mental health of oncology HCPs, as well as a limited set of recommendations on how to improve this situation. In the section that follows, we outline some of these knowledge gaps and offer some recommendations on how to improve the mental health of oncology HCPs during the ongoing pandemic and what will follow thereafter.

### 7.2. Increase Research on the Mental Health of Oncology HCPs

In our review, we found almost no studies that looked at the mental health disorders of oncology HCPs prior to the pandemic. Research that focused on the pandemic mostly focused on anxiety [50,52,58,71], but did not go beyond to the explore the psychological or emotional well-being as well as other specific relevant psychiatric disorders such as PTSD [68] and substance use of these healthcare professionals. Almost no studies pre- and post-pandemic have looked at the prevalence and risk factors for suicide among oncology HCPs despite data showing that physicians in other disciplines are at an increased risk for suicidality [75]. The limited data that do exist document concerning trends among oncology HCPs including elevated anxiety, depression, alcohol, and substance use as well as increased risk for suicide [35,36,38]. Data during the pandemic show an elevation in the mental health risks and prevalence of disorders [56,58,62,63,64,65].

However, more research is needed to understand the severity of mental health distress as well as delineate possible nuances in social and psychological factors contributing to the elevated risk to psychiatric morbidity and suicidality. Such research can increase awareness of the mental health status among oncology HCPs, battle the stigma associated with mental health (by substantiating it as a collective problem rather than a personal one), and can inform personal and structural interventions to mitigate it.

### 7.3. Eliminate Stigma and Shame about Mental Health Distress among HCPs

In a recent opinion piece in the *New York Times*, a physician noted “The culture of medicine discourages doctors like me from crying, sleeping or making mistakes. Worse, we can even be punished for seeking mental health care” [76]. This physician is correct in noting that there are severe consequences for medical professionals in seeking out mental health care that include the possibility of losing ones’ medical license, being subject to handing over personal medical records, and/or drug testing [77]. In a recent commentary in the *New England Journal of Medicine*, Arnold-Forster [77] and colleagues note that mental health is seen as a medical issue by healthcare professionals, and thus, considers physicians who have mental health or substance use problems to be ‘sick’. Moreover, the authors note that the focus on individual responsibility assumes that the HCP is personally accountable for their own wellness and that their inability to do so is their own fault [77]. The combination of these two core beliefs leads many HCPs to feel a deep sense of shame, isolation, and fear when they are experiencing mental health distress that discourages them from seeking the care they need [77]. This stigma has likely been exacerbated by the COVID-19 pandemic and it is highly likely that many oncology HCPs are suffering in silence and have not sought out the care they require [76]. This stigma can be reduced by implementing both macro- and micro-interventions. On a larger scale, physician licensing processes should not discriminate against HCPs with histories of mental health issues or those who are currently seeking mental health care [77]. On a smaller scale, as with burnout interventions noted above, mental health support for HCPs working in oncology should be part of the occupational health services available either through their own institutions [77] or through the provision of benefits where they can seek their own support without feeling a sense of stigma, shame, or fear of losing their licenses.

### 7.4. Implement Interventions That Address the Whole HCP

Our review of the literature revealed that some oncology HCPs developed a changed outlook on work–life balance as a result of the pandemic [50,51,62]. HCPs working in oncology settings reported wanting more time off and more flexible work schedules to accommodate their families. It is clear that the pandemic will have reverberating effects on the healthcare field for decades to come, particularly, as it relates to the desire of many HCPs to lead more balanced, heathy and family-friendly lives. The pandemic brought into sharp relief the need for family friendly policies that take into consideration the needs of HCPs with children. Interventions designed to enhance the quality of life of HCPs in oncology settings will have to include the provision of on site childcare and/or funds to help HCPs pay for childcare services. Other interventions include more time off from work, the ability to have more control over one’s schedule, and more flexible scheduling allowing for part-time or dynamic work schedules.

### 7.5. Considering Gender

Finally, related to the point above, our review revealed that almost no studies we found in our review looked at gender or childcare responsibilities as a factor in the impact of the pandemic on the mental health of oncology HCPs. This is a remarkable oversight as it has been robustly documented that the pandemic has had a disproportionate impact on working mothers, including in medicine [78,79,80,81,82,83,84]. For example, one study that looked at the impact of the pandemic on male and female oncologists found that in a sample of 541 oncologists, females were much more likely to report that the pandemic affected their professional career in a negative way (85%) when compared to males being asked the same question (76%). Women also reported that the COVID-19 pandemic affected their personal life (89% vs. 78% of men) and family life (84% vs. 77% of men) in negative ways and that they spent less time on science (39% vs. 25% of men) [60]. In order to design the best interventions and to truly address the components of mental health among oncology HCPs, gender will have to be taken into account. 

## 8. Conclusions

The global health crisis of the COVID-19 pandemic is far from being over. This crisis did not create the challenges that oncology HCPs face in their daily work, but the pandemic has clearly aggravated them. Research from the earlier stages of the pandemic documents the severe impact of the crisis on the mental health of HCPs in oncology settings. The high rate of burnout and added stress associated with the uncertainty and health risks associated with the management of the pandemic as a frontline caregiver have contributed to what has been coined as the ‘great resignation’ [85]. As a field, oncology can no longer ignore the mental health needs of HCPs. Interventions designed to reduce mental health distress and enhance the quality of life of HCPs in oncology settings will have to go beyond individual level self-care interventions (i.e., mindfulness interventions) to include the provision of structural changes and support (i.e., on site- childcare, flexible schedules etc.) [86]. Such structural interventions can include the implementation of family friendly policies, expansion of professional guidelines, and institutional protocols that include structures and processes to support HCPs in effectively coping with the stressors and losses that are part of their daily work. Battling the stigma that is associated with mental health concerns should be a priority. Structural efforts to combat it should include interventions to raise awareness and mental health literacy [87,88], as well as changing professional guidelines related to licensure to remove the sanctions associated with the reporting of mental health problems.

## Figures and Tables

**Table 1:** Mental Health of Oncology HCPs *Prior to* the COVID-19 Pandemic.

Burnout Compassion fatigue Secondary traumatic stress Grief over patient loss Depression and anxiety Excessive use of alcohol Use of sleep medications Moral distress and moral injury

**Table 2:** Impact of COVID-19 Pandemic on Oncology Healthcare Professionals’ Mental Health.

Changes at Work	Decrease in face-to face interactions with patients Deployment to other areas of the hospital (including frontlines) Lack of access to personal protective equipment Cancellation of surgeries and other treatments Increased workload Loss of autonomy Reduced job security Reduced income Reduction in research activities Transition to modifications in workflow schedules
Changes at Home	Children at home doing online school and the need to manage child’s education Family separation due to fear of contagion Decrease in quality of familial relationships Reduced time for family Reduced ‘personal time’ Negative impact on personal relationships with spouse and children Increase in domestic duties, particularly for women
Mental Health Distress	Increased mental health distress including depression and anxiety Feelings of a lack of control Feelings of uncertainty Guilt over not caring for patients and families Irritability Anger Post-traumatic stress symptoms Sleep disturbances Increased use of substances such as antidepressants, anti-anxiety medications, and sleeping medications
	Substantially increased rates of burnout
	Increased moral distress and moral injury
